# Assessment of Paroxetine Molecular Interactions with Selected Monoamine and γ-Aminobutyric Acid Transporters

**DOI:** 10.3390/ijms22126293

**Published:** 2021-06-11

**Authors:** Magdalena Kowalska, Łukasz Fijałkowski, Alicja Nowaczyk

**Affiliations:** Department of Organic Chemistry, Faculty of Pharmacy, Collegium Medicum in Bydgoszcz, Nicolaus Copernicus University, 2 dr. A. Jurasza St., 85-094 Bydgoszcz, Poland; magda.kowalska@doktorant.umk.pl (M.K.); l.fijalkowski@cm.umk.pl (Ł.F.)

**Keywords:** paroxetine (PRX), antidepressant, monoamine transporters (MAT), γ-Aminobutyric acid transporter (GAT), neurogenesis, monoamine and GABA neurotransmitters

## Abstract

Thus far, many hypotheses have been proposed explaining the cause of depression. Among the most popular of these are: monoamine, neurogenesis, neurobiology, inflammation and stress hypotheses. Many studies have proven that neurogenesis in the brains of adult mammals occurs throughout life. The generation of new neurons persists throughout adulthood in the mammalian brain due to the proliferation and differentiation of adult neural stem cells. For this reason, the search for drugs acting in this mechanism seems to be a priority for modern pharmacotherapy. Paroxetine is one of the most commonly used antidepressants. However, the exact mechanism of its action is not fully understood. The fact that the therapeutic effect after the administration of paroxetine occurs after a few weeks, even if the levels of monoamine are rapidly increased (within a few minutes), allows us to assume a neurogenic mechanism of action. Due to the confirmed dependence of depression on serotonin, norepinephrine, dopamine and γ-aminobutyric acid levels, studies have been undertaken into paroxetine interactions with these primary neurotransmitters using in silico and in vitro methods. We confirmed that paroxetine interacts most strongly with monoamine transporters and shows some interaction with γ-aminobutyric acid transporters. However, studies of the potency inhibitors and binding affinity values indicate that the neurogenic mechanism of paroxetine’s action may be determined mainly by its interactions with serotonin transporters.

## 1. Introduction

Thus far, modern psychiatry classifies many types of depression including episodic, chronic, post-schizophrenic, endogenous, exogenous and other [1]. To explain the cause of depression a variety of hypotheses have been proposed. These include the monoamine hypothesis, neurogenesis hypothesis, neurobiology hypothesis, inflammation hypothesis, and the stress hypothesis [2,3,4]. The monoamine hypothesis suggests a relationship between a patient’s depressed mood and a decrease in the level of selected neurotransmitters. Until now, over 100 different neurotransmitters have been discovered [5]. It is widely accepted that in depressive states the key neurotransmitters are serotonin (SER), noradrenaline (norepinephrine) (NE), dopamine (DA) [6,7,8] and γ-aminobutyric acid (GABA) [9,10,11]. SER, NE and DA contain a catechol moiety, and thus belong to catecholamines, known as excitatory neurotransmitters. GABA, on the other hand, belongs to the group of acidic neurotransmitters and is the main inhibitory neurotransmitter in the brain. The role of controlling neurotransmitter levels has been confirmed by the discovery of antidepressants such as tricyclic antidepressants (TCA’s), selective serotonin reuptake inhibitors (SSRI’s) and serotonin norepinephrine reuptake inhibitors (SNRI’s) that increase serotonin levels in the brain [4]. Many of the modern generations of antidepressants act as inhibitors of one or several of the monoamine transporters (MATs) [12,13] and/or GABA transporters (GAT) [14]. The neurotransmitter transporter family dependent on sodium and chloride (SLC6 transporters) and including norepinephrine transporter (NET), dopamine transporter (DAT), serotonin transporter (SERT) and GABA transporters (GATs), encoded by SLC6A1-4 genes in humans are specifically known to be important for efficient neuronal synaptic transmission, hence providing neurotransmitter homeostasis in the CNS [15]. Briefly, inhibition of MATs/GATs leads to reduced clearance of neurotransmitters after synaptic release, thus increasing the neuronal signal intensity [11,16,17]. The inhibitors act by increasing the amount of neurotransmitters in the synapse, and in consequence alleviate or subside symptoms of depression [18].

Currently, one of the most commonly used antidepressants is paroxetine (PRX, (3S,4R)-3-([benzo[d][1,3]diokso-5-iloksy]metylo)-4-(4-fluorofenylo)piperidina), which belongs to the SSRI group [19]. It effectively increases the concentration of endogenous SER in the synaptic cleft [20,21,22]. It is noteworthy that it usually takes several weeks to achieve the desired antidepressant therapeutic effect of PRX. This therapeutic delay suggests that slow adaptive changes in the neural circuits during long-term pharmacological treatment possibly involve changes in gene expression and protein translation [23,24]. This in turn supports the hypothesis that depression is caused by disruption of functional and structural connections of the neural circuits that underlie the regulation of mood. However, the detailed molecular mechanisms of PRX have not been fully recognized and explained so far.

The studies presented in this publication are intended to answer the question about the possibility of PRX acting in the neurogenic mechanism. The primary purpose of this study is to analyze thoroughly the interactions between PRX and human MAT (hMAT: hSERT, hDAT, hNET), human GAT (hGAT: hGAT1-3, hBGT1). According to an extensive literature survey we have established that, however, the literature is replete with different studies concerning the four key transporter proteins, and there is a lack of pharmacological data on the molecular level. Therefore, we have undertaken the effort to fill this gap. In order to achieve the goal of this study we have conducted the in silico research involving dual directional analyses of the issue. The first mode of the analysis was designed to compare the analyzed ligand and protein structures by means of superimposition procedures. The results of this part of study delivered information about the structural similarities, which are often used in the pharmacological preliminary studies. The second part of analysis comprised a detailed inquiry of the intermolecular interactions present in the complexes especially hydrogen bonding. To compare the binding strength of PRX with individual transporters, the following reference compounds were selected, SER, DA, NE and GABA. The interactions were investigated by means of docking studies of selected antidepressant compounds performed on the crystal structure of hSERT [25] and on homology modeled hNET [26], hDAT [27] and hGAT1 [28]. It is noteworthy that the sequence identity between the template (i.e., sequences with known 3D structure) and the modeled sequence is 63.3% for hNET, 52% for hDAT [29], and 47% for hGAT1 and to the best of our knowledge this is the highest available identity. Reaching a general aim of the study requires also pharmacological investigation to provide the reader with experimental context. Even though the literature study shows that PRX–MAT interactions are extensively investigated by means of pharmacology, there are still gaps in the assessment of PRX–hGAT interactions. The pharmacological part of the present study includes the presentation of PRX–hGAT data that helps to understand and assess the neurogenesis hypothesis. This part of the study covers the investigation of the inhibitory effect of PRX, SER, NE and DA at the four hGATs (hGAT1-3, BGT1) using the [^3^H]GABA uptake assay.

## 2. Results

### 2.1. Molecular Docking Studies

#### 2.1.1. Structural Similarity Studies for Neurotransmitters and Their Transporters

**Figure 1:**
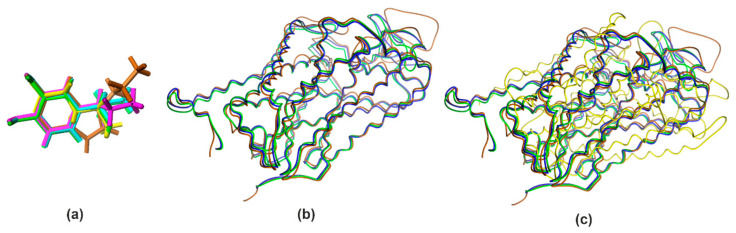
Superimposition of the investigated docked structures of neurotransmitters: serotonin (SER, brown), dopamine (DA; blue), noradrenaline (NE, green), γ-aminobutyric acid (GABA, yellow) (**a**), and human monoamine transporters: serotonin transporter (hSERT, brown), dopamine transporter (hDAT, blue), norepinephrine transporter (hNET, green) (**b**), human transporters: serotonin transporter (hSERT, brown), dopamine transporter (hDAT, blue), norepinephrine transporter (hNET, green), and γ-Aminobutyric acid transporters 1 (hGAT1, yellow) (**c**).

The Tanimoto similarity coefficient (Tsc) for pairwise comparison of molecules is probably the most widely used estimator of molecular similarity. This parameter by definition ranges from 0.0 (completely different) to 1.0 (equal molecules) [30]. Results of 2D similarity tests can be arranged in descending order of the Tsc: Tsc_DA/NE_ = 0.59, Tsc_SER/DA_ = 0.46 Tsc_SER/NE_ = 0.35, Tsc_SER/GABA_ = 0.25, Tsc_DA/GABA_ = 0.12, Tsc_GABA/NE_ = 0.05. They show a large structural diversity of the studied neurotransmitters, among which the greatest similarity of DA was noted to NE and SER. To obtain additional information concerning the shape of the investigated neurotransmitters and transporters, the active conformations were chosen for superimposition. The atoms common to these molecules were selected for the fitting procedure. Their similarity was calculated as RMS fit. The RMS routine provided estimates of how closely molecules fit to each other. The lower the RMS value, the better the similarity. The docked structures of the studied molecules were compared by their superimposition using a least-squares algorithm that minimizes the distances between the corresponding non-hydrogen atoms as shown in Figure 1.

The analysis of similarity of obtained active structures shows that all four compounds adopt very similar conformations in the target site of the protein RMS ≈ 0.1–1.5. The only significant deviation of the structure concerns the alkyl regions.

Recent studies on the identification of homologous structures for the studied proteins indicate that the binding site of hSERT shares a sequence identity of 57% with hDAT and 68% with hNET. Additionally, the identity between hNET and hDAT is 86% [31]. The hDAT shows the greatest homology to hNET within the binding site with an amino acid sequence identity of 78% [32,33]. The homology for each human transporter related to the others was sequence identified is approximately hDAT/hNET = 67%, hDAT/hSERT = 50%, hNET/hSERT = 53%. The evidence from these studies presents the lower sequence similarity for hNET/hDAT = 58% [34] compared with hGAT1/dDAT = 66% [35] of identity. All these values are quite similar to the sequence of proteins in all the transporters analyzed. By definition the percentage of structurally equivalent positions was defined as the percentage of the two alpha carbon atoms in the shorter of the sequences that are less than 3.5 Å of the equivalent atoms in the superposed structure [36]. All these values indicate a significant similarity in the sequence of proteins in all transporters analyzed (Figure 1). However, the analysis of structural similarities counted as RMS (in Å) superimposition the structures of two proteins [37], with alpha carbons of corresponding amino acids being taken together [38,39], results for MAT as following data for hSERT: hSERT/hDAT = 5.70 Å, hSERT/hNET = 5.66 Å. These results indicate a comparable similarity of both studied pairs, with a slightly higher similarity between hSERT and hNET than hSERT and hDAT. On the contrary, the obtained RMS value for hSERT/hGAT1 = 19.62 Å pair clearly indicates a significantly different 3D structure of the protein analyses. Similar RMS values were observed for hGAT1 with the MAT structures studied: hGAT1/hSERT = 19.62 Å, hGAT1/hNET = 19.82 Å, hGAT1/hDAT = 19.6 Å. These data indicate a low probability of strong interactions between PRX and hGAT1, while it is likely that a better interaction of PRX is seen with hNET and hDAT although hNET seems to have a slightly better interaction compared to hDAT. Furthermore, despite sharing high sequence identity and a similar MAT-structural fold (Figure 1), the pharmacology of neurotransmitter transporters is diverse, primarily because of the amino acid variations in the central binding site [40].

#### 2.1.2. hSERT

Based on many pharmacological studies previously presented in the literature, PRX has been classified as an SSRI due to its very high inhibitory potencies for SERT. On the basis of predicted binding affinity, the highest value was observed with PRX (calculated pK_ihSERT_ ≈ 10.20, Table 1) compared to all studied compounds and hSERT.

**Figure 2:**
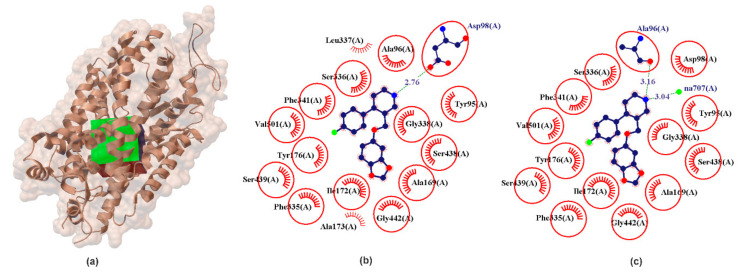
X-ray structure of the hSERT crystal (**a**) with the grid box showing the ligand-binding site (PDB ID: 5i6x, 3.14 Å). Comparison between calculated (**b**) and crystallographic data (**c**) for binding pocket in PRX–hSERT complex. The red circles and ellipses identify equivalent residua (in 3D superposition of structures).

In various studies, pIC_50SERT_ was found to be between 8.96 and 10.40 [16,21,41,42,43]. This is in agreement with our docking results that shows that the most stable complex is for hSERT-PRX (E_B_ ≈ 13.92 kcal/mol). The second strongest interaction with hSERT was SER (calculated pK_iSERT_ ≈ 7.11, E_B_ ≈ 9.7 kcal/mol, Table 1). The results of the present study demonstrate that DA, R/S-NE and GABA have low stability (E_B_ ϵ (3.86; 3.99 kcal/mol). Other studied hSERT-neurotransmitters complexed have calculated pK_i_ value ≤ 4 indicating weak ligand/inhibitor binding affinity. Comparing these values to other currently used antidepressants (pIC_50SERT_ ≈ 9.10; 9.54; 8.05; 8.92; for fluoxetine (Prozac) [41], sertraline (Zoloft) [16], citalopram (Celexa) and venlafaxine (Effexor) [16], respectively) confirm the fact that PRX is the strongest SERT inhibitor currently known. Additionally, it is stronger than SER (pIC_50SERT_ ≈ 7.00 [21,43]). Given the extraordinary strength with which PRX inhibits hSERT, the study of molecular factors that influence the neurogenesis phenomenon seems warranted. It is worth highlighting that in the biological activity under consideration, hSERT is the only transporter studied whose crystallographic structure is known (PDB ID: 5i6x, 3.14 Å [25]). The localization of PRX binding sites in crystals is well understood and described [19]. It is a central substrate binding pocket, commonly located approximately halfway across the membrane bilayer and marketed as S1 pocket (Table 2, Figure 2).

From chemical point of view hSERT is composed of three different regions; A, B, C. Subsite A is defined by Tyr95, Ala96, Asp98, Gly100 form transmembrane segments helix 1 (TMH1), and Phe335, Ser336, Gly338, Phe341, Val343 from transmembrane segments helix 6 (TMH6) and Ser438, Thr439, Ala441, Gly442 from transmembrane segments helix 8 (TMH8) and is define as a polar region surrounding Asp98. In crystallographic data it was found that regions A accommodates the polar, amine moiety of the PRX which form one hydrogen bond between NH_2_ group and Ala96 (Figure 2). Subsite B is the groove delineated between Ser438, Thr439, Ala441, Gly442 (TMH8) and Ala 169, Ile172, Ala173, Tyr176 from transmembrane segments helix 3 (TMH3). It has been confirmed by X-ray research that PRX locates a benzodioxol group in this groove. Subsite C is defined as a region between TMH6 and TMH10 (Val489, Lys490, Glu493, Glu494, Thr497, Gly498, Pro499, Leu502) and is located in the extracellular vestibule. Subsite C can interact with bulky drugs. Structurally B region is located opposite to subsite C and both are largely hydrophobic regions [46]. Commonly the pose obtained from X-ray diffraction is often denoted by many authors as ABC [22]. The results obtained in our PRX docking and validation experiments also remain to indicate PRX orientations in the ABC pose. In our experiment, the amine group of piperidine ring donates one hydrogen bond to the oxygen of the carboxylic moieties of Asp98 (Figure 2) with E_HB_ ≈ −1.68 kcal/mol and bond length of 2.76 Å. The docking experiment confirms that the PRX molecule has a hydrophobic effect with an identical set of amino acids compared to crystallographic data (Figure 2, red circles and ellipses identify equivalent residua (in 3D superposition of structures)).

All calculated molecular configurations indicate that the energy of the single hydrogen bond between studied compounds and the set of neurotransmitters is relatively low (Table 1). The highest hydrogen bond energy is found for the major hydrogen bond component of Gly65.

#### 2.1.3. hDAT

hDAT is the major target of addictive psychostimulants such as cocaine, which bind to the active site and prevent the conformational transition of the transporter, thereby inhibiting the reuptake of dopamine [40]. DAT mediates reuptake of DA (pIC_50SERT_ << 4, pIC_50NET_ ≈ 5.28, pIC_50DAT_ ≈ 5.06 [21] − 5.61 [32]) from the synaptic cleft and thereby controls the termination of dopaminergic signaling.

**Figure 3:**
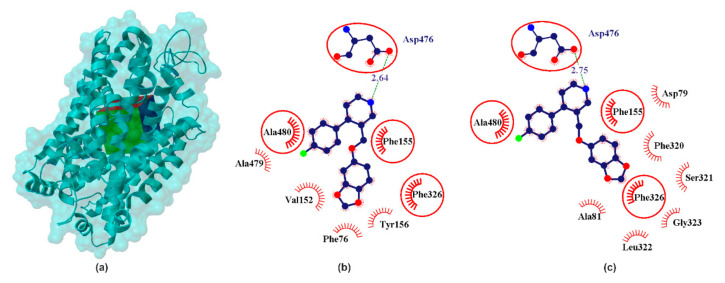
X-ray structure of the hDAT homology model (**a**) with the grid box showing the ligand-binding site (Swiss-Prot ID: Q01959) and calculated data for cocaine binding pocket (**b**) and clomipramine binding pocket in PRX–hNET complex (**c**). The red circles and ellipses identify equivalent residua (in 3D superposition of structures).

The DAT structure distinguishes binding places defined as hDAT/cocaine (pocket 1) and hDAT/clomipramine (pocket 2) [6]. Cocaine is a high-potencies inhibitor of DAT (pIC_50SERT_ ≈ 6.54, pIC_50NET_ ≈ 5.48, pIC_50DAT_ ≈ 7.14 [47]) and it is thought that its binding to DAT causes a rapid increase in extracellular dopamine levels [48] that produce the reinforcing effects leading to cocaine abuse. In addition, cocaine, despite the increase in DA in the synapse, does not act as an antidepressant. As confirmed in many studies, cocaine abuse and addiction are associated with an increased risk of depression [49]. Binding site for DA and cocaine in DAT overlap and are named by many authors as the central binding site, surrounded by TMs 1, 3, 6, 8 [33,50]. Clomipramine is a tertiary amine belonging to a dibenzazepine TCA [51]. Clomipramine has a stronger potency for the serotonin transporter (pIC_50SERT_ ≈ 9.52, pIC_50NET_ ≈ 7.42, pIC_50DAT_ ≈ 5.66 [16]), compared to other TCAs [51]. Clomipramine binding site in hDAT protein is surrounded by TMHs 1, 3, 6, 10 and 11 [6]. Thus, two binding pockets have particular interest for the design of novel DAT interacting ligands. As shown in Figure 3 in both pockets PRX forms a hydrogen bond with Asp476. Comparing hydrophobic interactions in the analyzed cases reveals partial interaction compatibility (Figure 3, red circles identify equivalent residua (in 3D superposition structures)) but it also points to other residua in both cases (Figure 3, red arcs identify residua in hDAT/cocaine and hDAT/clomipramine, respectively (in superposition 3D structures)). The resulting energy values (Table 1) indicate that PRX forms a more energy-stable complex in the cocaine pocket. It is noteworthy that all neurotransmitters analyzed interact with the hDAT transporter (Table 1). It seems somewhat astonishing that SER interacted more strongly with hDAT (pIC_50DAT_ ≈ 5.57 [21]) than the DA itself (pIC_50DAT_ ≈ 5.06 [21]). Racemate R/S-NE, on the other hand, has a potency of pIC_50DAT_ ≈ order of 4.28 [21]. However, this is confirmed by literature data.

#### 2.1.4. hNET

**Figure 4:**
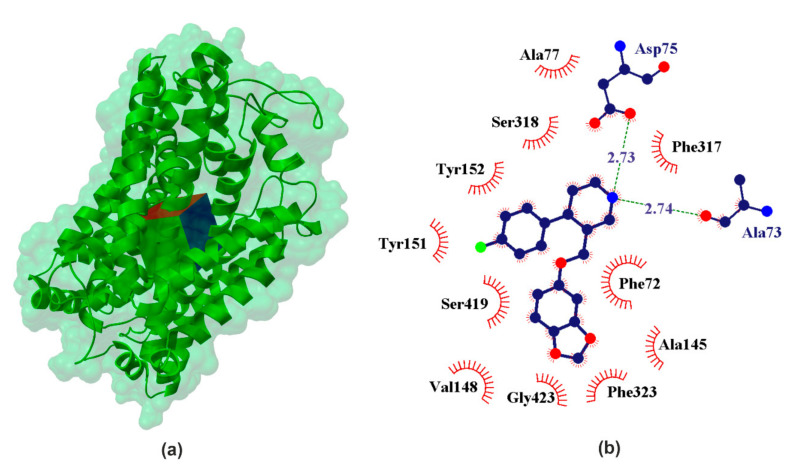
X-ray structure of the hNET homology model (**a**) with the grid box showing the ligand-binding site (Swiss-Prot ID: P23975) and calculated data (**b**) for binding pocket in PRX–hSERT complex.

Norepinephrine (also called noradrenaline) is a neuromodulator that in multiple ways regulates the activity of neuronal and non-neuronal cells [52]. It was one of the first neurotransmitters to be discovered [53]. It was isolated in 1901 and is the first hormone obtained in the crystalline state [54]. The presence of norepinephrine in two main enantiomers (R/S-NE) was confirmed by structural (crystallographic and spectroscopic studies [54,55]). In crystallographic study it was proved that NE has a zwitterionic structure, formed by proton transfer from the meta phenolic hydroxyl group to the nitrogen atom (^−^O(OH)Ph-CH(OH)-CH_2_-NH_3_^+^) [56]. It was founded approx. 6.5–7.7 Å due to separation of charged centers [55], which determines the molecule of the interaction with neurons [28]. These conformers are stabilized by intramolecular hydrogen bonds between the nitrogen atom of the amino group and hydrogen of hydroxyl group attached to an ethyl amine carbon atom [55]. NE is the second major biogenic amine that has been proposed to be causally involved in the pathophysiology of major depression and in the mechanism of antidepressant drug action. The norepinephrine transporter (NET) is a transmembrane protein responsible for transporting norepinephrine into the synaptic terminals of the central and peripheral nervous systems as well as neuroendocrine adrenal chromaffin cells [57]. The selective NE reuptake potencies inhibitor, reboxetine (pIC_50_ ≈ 8.96 [16]), has demonstrated equivalent efficacy to the TCA in some studies and is approved as an antidepressant in Europe but not in the USA [58] (Figure 4).

The inhibitory potencies R/S-NE for its transporter lie in the range pIC_50NET_ ϵ (6.17 [32]; 5.06 [21]). Comparison of the data obtained for our docking experiment (Table 1) clearly leads to the conclusion that S-NE shows stronger binding affinity than the other studied neurotransmitters. It was found in our experiment that pK_iNET_ ≈ 5.59 for S-NE and pK_iNET_ ≈ 4.12 for R-NE. Another strength of the interacting neurotransmitter is DA (calculated pK_iNET_ ≈ 5.31), which is confirmed by the literature data (pIC_50NET_ ≈ 5.28 [21]). Among the studied catecholamines, serotonin is characterized by the weakest affinity for NE transporters (calculated pK_ihNET_ ≈ 4.33 and pIC_50NET_ ≈ 4.85 [21]). Calculated binding affinity pK_ihNET_ ≈ 3.31 for GABA suggests interaction with hNET. These data confirm that NET has some selectivity relative to NE. When it comes to the effects of PRX on NET, it is much stronger (calculated pK_ihNET_ ≈ 7.61 and pIC_50NET_ ϵ 6.46 [21] − 7.40 [16]) compared to all the neurotransmitters in the studied set.

#### 2.1.5. hGAT1

**Figure 5:**
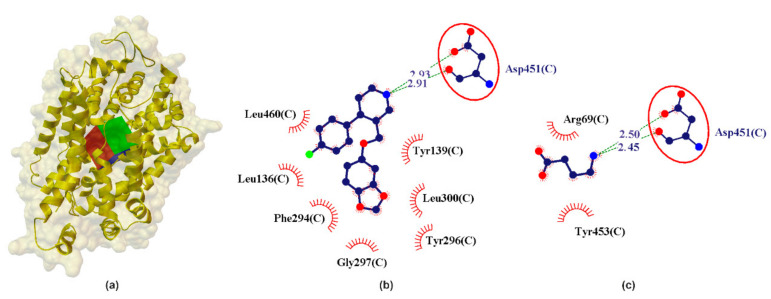
X-ray structure of the hGAT1 homology model (**a**) with the grid box showing the ligand-binding site (Swiss-Prot ID: P30531) and calculated data (**b**) for binding pocket in PRX–hGAT1 and (**c**) GABA–hGAT1 complex. The red circles and ellipses identify equivalent residues (in 3D superposition of structures).

In regions such as the cerebral cortex, hippocampus, thalamus, basal ganglia, cerebellum, hypothalamus, and brainstem, GABA represents about one-third of the synapses. GAT1 is the most copiously expressed GAT in the CNS and is mainly localized into the presynaptic axon terminal and into a few astrocytic structures [59]. The GABA chain is made of three single carbon-carbon bonds, thus its structure is highly flexible. This determines the ability to adapt various conformations that determine binding, as ligand, in the proteins with which it interacts. Crystallographic studies have shown that both crystal and aqueous solution of GABA occur in the form of a zwitterion, which in the body makes it a carrier of electric current and thus creates the possibility of carrying stimuli. It is widely accepted that distance between cationic and anionic center approx. A value of 5 Å determines the neuronal and glial blocking effect in selective inhibitors. Computational methods have shown that in an aqueous solution more than 94% of GABA molecules adapt a folded conformation with separation of charged centers below 5 Å [28]. GABA is transported by GAT in a somewhat folded conformation (Figure 5). This means GABA interacts with neuronal and astrocytic structures.

In the preliminary analysis of the docking it was observed that GABA interacts with the active site of hGAT1 with calculated pK_ihGAT1_ ≈ 4.49 and [^3^H]GABA pIC_50hGAT1_ ≈ 5.00 [60,61]. The studied catecholamines show significantly less interaction with hGAT1 as compared to GABA (Table 1). It is noteworthy that PRX shows the strongest interactions with hGAT1 (calculated pK_ihGAT1_ ≈ 4.35) compared to SER, NE and DA. (Table 1). PRX creates a bifurcated hydrogen bond, similar to the GABA–hGAT1 complex, where NH_2_ from PRX is involved in a COO...NH_2_...CONH incorporating the COO from Asp451 and the NHCO from Asp451 (Figure 5). From the data in Table 1 it can be seen that this bifurcated system is almost geometrically (L_HB_ ≈ 2.92 Å) and energetically (E_HB_ ≈ −3.41 kcal/mol) symmetric, where the major hydrogen bond component is fixed into the side-NH-CO of Asp451 and the minor involves the side-COO of Asp451 residue (Figure 5).

#### 2.1.6. Validation Experiment

**Figure 6:**
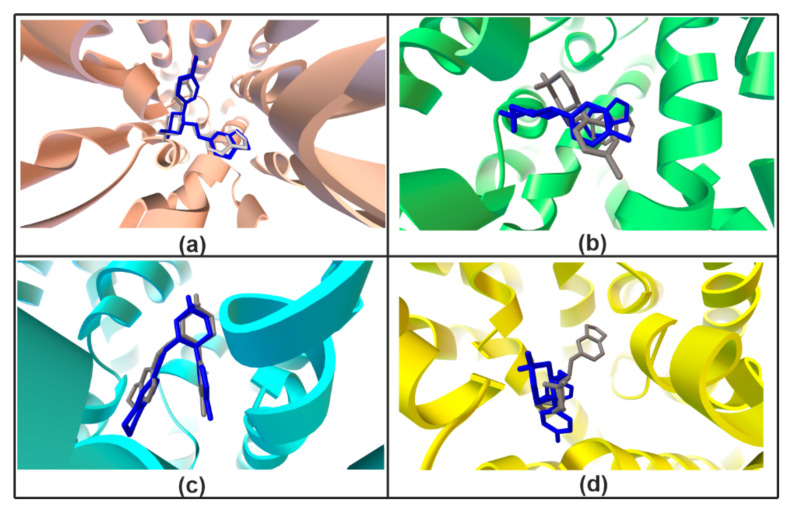
Validation redocking experiment. Docking results of the hSERT (**a**), hNET (**b**), hDAT/cocaine (**c**), hGAT1 (**d**) obtained using AutoDock (gray) and Vina (blue).

The re-docking method was applied to confirm legitimacy of the docking procedure [62] and was performed using Vina software [63]. Results of redocking in Vina (optimal poses) were saved as pdbqt files. Subsequently, the AutoDock Tools (ADT) program was employed to calculate the superimposition of the ligand and protein complexes. The validation process indicates a high similarity of PRX corresponding poses obtained using AutoDock and Vina (Figure 6).

### 2.2. Inhibitory Activities of PRX, SER, NE and DA at hGATs

**Figure 7:**
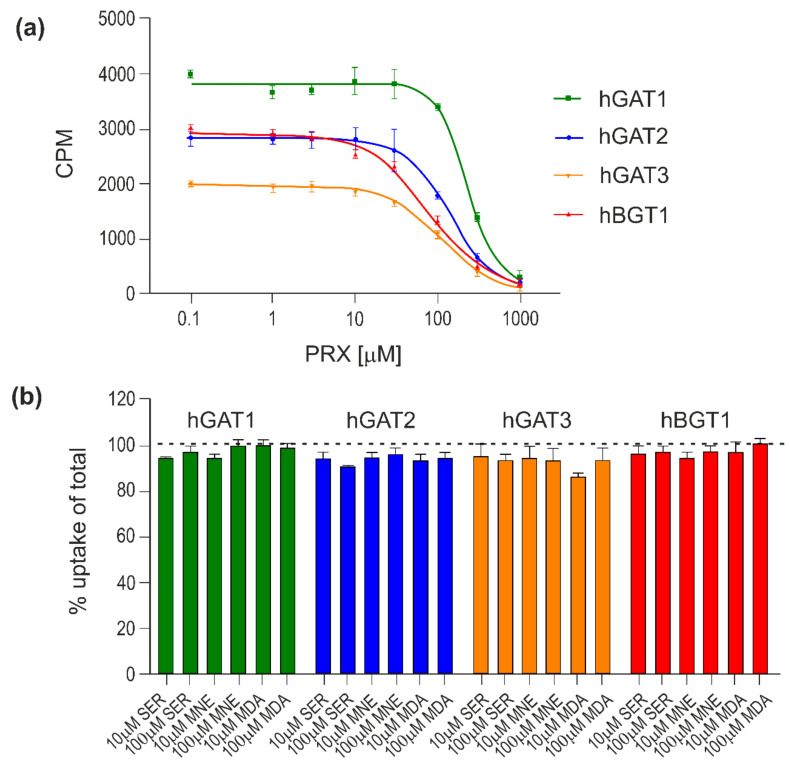
Inhibitory activity of PRX, SER, NE and DA at hGATs. The compounds were tested for their ability to inhibit the uptake of 30 nM [^3^H]GABA for 3 min at all hGATs stably expressed in CHO cells. Representative concentration–response curves of PRX at hGAT1-3 and hBGT1 (**a**) and inhibitory activity of SER, NE and DA at hGAT1-3 and hBGT1 (**b**). All experiments were performed with technical replicates in three independent experiments and depicted as means ± S.D in (**a**) or normalized means ± S.E.M (**b**).

PRX concentration-dependently inhibits the [^3^H]GABA uptake mediated by all four hGATs with mid-high micromolar potency in the range of 85.6–256.1 µM with the rank order: hBGT1 < hGAT3 ≈ hGAT2 < hGAT1 (Figure 7a and Table 3).

SER, NE and DA display limited inhibitory activities at hGAT1-3 and hBGT1 with less than 10% inhibition observed at compound concentrations up to 100 μM (Figure 7b).

Structurally PRX can be thought of as a substituted derivative of piperidine where in position 3 there is a group of benzodioxol and in position 4 the fluorophenyl group. The closest to PRX active structure in hGAT is nipecotic acid, which belongs to the leading structures for the development of structural hGAT inhibitors and has the following inhibition potential: pIC_50hGAT1_ ≈ 4.7, pIC_50hGAT2_ ≈ 3.3, pIC_50hGAT3_ ≈ 4.0, pIC_50hBGT1_ ≈ 2.5 [61]. A comparison of the inhibition potential of PRX (Table 3) and nipecotic acid indicates that the replacement of the carboxyl group with benzodioxol and the addition of the fluorophenyl group change the activity profile from hGAT1 and hGAT3 to hGAT2, and hBGT1. Consequently, one can draw a preliminary conclusion that such a modification of the structure makes the molecule more susceptible to inhibition of hGAT located outside the CNS. Which in turn indicates that PRX has less influence on the neurogenesis process in the brain.

## 3. Discussion

Decreased amounts of SER, NE, DA and GABA have been observed in people with depression. Increasing SER in key CNS pathways and at desired serotonin receptor subtypes hypothetically mediates therapeutic actions in depression, and other diseases with similar symptoms such as OCD, PD and bulimia [64]. This is the result of the powerful influence of SER on emotionality that has been proven many times over [65]. SER and GABA are mainly, but not only, the basic inhibitory neurotransmitters in the CNS. They play an important role in processing neuronal information as well as regulating neurogenesis (proliferation, differentiation and migration of neural stem cells (NSC)) [66,67]. Numerous studies have shown that deficits in serotonin, norepinephrine and GABAergic neurotransmission and reduced neurogenesis are associated with the ethology of pathological anxiety and various mood disorders including depression [67,68,69]. Evidence of reduced neurogenesis has been observed in animal models of depression and in postmortem studies of individuals who had been diagnosed with major depressive disorder. However, the mechanisms driving these alterations in neurogenesis are not fully understood [70]. SER, NE and GABA have been shown to regulate hippocampal neurogenesis and neuronal development in both children and adults [71,72]. In the adult brain, as in the embryonic nervous system, SER, NE and GABA depolarizes neural progenitors and immature neurons [73]. GABA also regulates quiescence of NSCs, differentiation of NSCs into neural progenitor cells (NPCs), maturation of NPCs into granule cells and synaptic integration of adult-born granule cells into the existing circuitry of the hippocampus [72]. A common feature of depression and anxiety disorders are SER, NE and GABA deficits resulting in emotional control disorders [65]. Recently, a direct link between chronic antidepressant treatment and an enhancement of SER, NE and GABA transmission were found [65,67]. This is in fact that SER, NE and GABA concentration are reduced in cortical brain and CSF in major depression, but its deficit can be reversed by chronic SSRI and electroconvulsive therapy [74,75].

PRX is an antidepressant drug known by the commercial names Aropax, Paxil, Pexeva, Seroxat, Sereupin and Brisdelle. It exhibits the highest known inhibitory potency for the active site of the hSERT (Table 1). A plethora of publications devoted to PRX (e.g., PubMed database 1992–2021 the word “paroxetine” indicates in about 6200 publications, Science Direct about 22000, Google Scholar about 54,500 works) proves the great importance of this drug in current medical therapies. These works provide a lot of information on the different aspects of taking this medicine and its mechanisms of action and it is clear that PRX is currently clinically approved for the treatment of numerous neurological disorders and not only depression [22].

After administration of PRX the level of SER rapidly increases (after a few minutes), while the antidepressant effect does not appear before after several weeks of chronic treatment [18,53,67,76]. This may indicate a possible different mechanism of action of PRX, and the most probable seems to be the neurogenic mechanism of action [77]. For this reason, we undertook studies of PRX interactions with four basic transporters, such as three MAT and GAT, the inhibition of which is crucial for increasing the level of basic neurotransmitters in the brain. At the beginning using the results of research based on molecular modeling techniques, the structural similarity of studied neurotransmitters: SER, NE, DA and GABA, as well as their respective targets, i.e., MAT and GAT1 transporters were analyzed. Using various techniques, such as the Tanimoto coefficient [30] and superimposition tests [63], it was shown that some structural similarities were found within the tested neurotransmitters as well as their transporters. Nevertheless, the analysis of structural similarities calculated as RMS determined by superimposing the structures of two proteins seems to be of key importance in understanding the interaction of various compounds with the analyzed objectives [37]. These results, in contrast to the data routinely presented in the literature based on the amino acid sequence identity technique [29,32,33,34,35], better highlight the structural differences of the analyzed transporters. These differences are of particular importance within the ligand binding site. The information obtained in this study indicates some unsimilarities between MAT and GAT1 (Figure 1), which may explain the low inhibitors of potency and binding affinity values of PRX for GAT1-3 and BGT1. On the other hand, a more detailed analysis of the literature data on the interactions of PRX and nipecotinic acid (as one of the leaders for the hGAT inhibitor family) provides information about the direction of interaction of these molecules as substituted piperidine derivatives. The data we obtained based on the in silico and in vitro methods indicate that PRX interacts with each monoamine transporter and shows some interactions with the hGATs (Table 1 and Table 3, Figure 7). As shown in the Section 2, the obtained in silico data are confirmed by the relevant pharmacological studies for MAT previously published in the literature. It has recently been found that PRX may promote the proliferation of nerve cells in vitro, as well as hippocampal neurogenesis in human and animal models [78]. Nowadays it is well accepted that depletion of SER in the brain results in suppression of neurogenesis in the adult hippocampal. At the same time, raising levels of monoamine, serotonin and norepinephrine and GABA increases the rate of neurogenesis. Since PRX is the strongest known hSERT inhibitor, the study of the possible mechanism of antidepressant action clearly points to issues related to the phenomenon of neurogenesis. However, the neurogenesis process itself is not yet sufficiently explained at the molecular level. Since this phenomenon is known to be linked to an increase in serotonin and norepinephrine and GABA levels within neurons, it seems that the potential for blocking suitable transporters is at the heart of this phenomenon.

It is important to emphasize that the interaction of PRX with the hGATs seem to be a more complex issue due to the multitude of different subtypes of hGAT. Hitherto four subtypes of plasma membrane human transporters for GABA (hGAT1–3 and hBGT1) have been identified [79,80,81], of which hGAT1 and hGAT3 have the highest expression levels in the mammalian central nervous system (CNS). hBGT1 is only found in scarce amounts and hGAT2 is not found in the brain parenchyma at all [82]. hGAT1 is predominantly located on GABAergic nerve terminals [35,59,83], while hGAT3 and hBGT1 are commonly associated with perisynaptic and distal astrocytic sites [28,60]. The location and level of individual transporters in CNS seem to be crucial in the neurogenesis process. Our experiments show that PRX interacts with all hGAT (Table 1 and 3 and Figure 7) to varying degrees. Interaction of PRX with individual hGATs can be arranged in descending order: pIC_50hBGT1_ ≈ 4.10, pIC_50hGAT3_ ≈ 4.00, pIC_50hGAT2_ ≈ 3.90, pIC_50hGAT1_ ≈ 3.6 (Table 3) and pK_ihGAT1_ ≈ 4.35 (Table 1). In vitro data clearly confirm the strongest interaction with hBGT1. However, due to the very low level of BGT1 expression in the brain (about 100–1000 times lower than GAT1 [84]), BGT1 can at most be responsible for 0.1–1.0% of the GABA transport. In addition, neurotransmitters diffuse rapidly out of the synaptic cleft on a low microsecond time scale until they bind to transporters and are removed [85,86,87], hence BGT1′s functional role in the neurogenesis process seems negligible. For these reasons and in light of the low inhibitory potency of PRX at hGAT1 and hGAT3, it appears that the interaction of PRX with the hGATs has little effect on the PRX induced neurogenesis process. Due to this analysis one can assume that if PRX induced neurogenesis it potentially connected with its inhibition to hSERT due to higher value inhibitory potency and binding affinity.

## 4. Materials and Methods

### 4.1. Pharmacological Studies

Flp-In Chinese hamster ovary (CHO) cell lines stably expressing hGATs (hGAT1-3 or hBGT1) used for the pharmacological studies have been described previously and were cultivated accordingly [88]. The [^3^H]GABA competition uptake assay was performed exactly as previously described [61], and the [^3^H]GABA uptake data were normalized to the percentage of total uptake in the individual experiments. Data presented are the pooled data of three independent experiments with three technical replicates. Concentration–response curves were fitted with GraphPad Prism (version 9.0.0, Yosemite, GraphPadSoftware, San Diego, CA, USA) as outlined previously [88].

### 4.2. In Silico Studies

#### 4.2.1. Docking Study

The calculation procedures applied in the study are typical for processing of docking studies [63,89,90,91].

##### Ligand Preparation

For the 3D molecular structure calculations, the Gaussian 09 (version D.01. for Unix/Linux) package was used [92]. The initial accepTable 3D structures of 6 studied compounds were downloaded (as mol2 file) from ZINC [93]. Later, the GaussView [92,94] was applied for preparation of Gaussian input files. All the molecules were geometry-optimized in water described by the PCM (polarizable continuum model). DFT/B3LYP level of theory, 6-311 + G(d, p) basis set. After geometry optimization (the root-mean-square gradient value smaller than 10^−6^ a.u.) compounds were saved as mol2 files using the GaussView. Subsequently, torsionals and number of active torsions for ligands were defined and the Gasteiger charges were assigned to each compound via AutoDockTools (ADT) [95]. Finally, ligands prepared for docking were saved as pdbqt files.

##### Monoamine and γ-Aminobutyric Acid Transporters Preparation

Despite numerous protein sequences in RCSB Protein Data Bank, lack of three-dimensional structures of many important drug targets is still observed. As a consequence, homology modelling is the different option to construct an accepTable 3D model of the protein. Therefore, in this study docking experiments were mostly performed on the homology modeled proteins (hDAT, hNET and hGAT1), except hSERT, where the X-ray structure is available.

- hSERT

The structure of the ts3 hSERT was gained from the RCSB Protein Data Bank (5I6X pdb access code) [25] First, the pdb structure was adapted for ADT software environment. Accordingly, one crystallographic water molecule and following ligands: dodecyl-beta-d-maltoside (LMT), cholesterol (CLR), Paroxetine (PRX), 2-acetamido-2-deoxy-beta-D-glucopyranose (NAG) were removed. Subsequently, missing hydrogen atoms were added and protein was saved in pdbqt format [96].

- hDAT, hNET hGAT1

As mentioned before, for hDAT, hNET and hGAT1 homology human protein sequences were obtained from the Swiss-Prot database (accession numbers: Q01959, P23975 and P30531, respectively). In this study the most actual homology modeled channels were used [6,26,44,97]. After that, pdb files were opened in ADT, which read coordinates. Subsequently, charges were added, correct atom types were assigned and nonpolar hydrogens were merged. Finally, according to the procedure the prepared protein was saved as a pdbqt file.

##### Molecular Docking

Preparation of the inputs for the modeling was carried out in full compliance with the applicable procedures. A grid box with a dimension of 60 × 60 × 60 Å^3^ and a grid spacing of 0.375 Å was built in the middle of the pore forming sequences. In this in silico experiment we consider the interaction between ligands and intracellular pore gate formed from the transmembrane helices. For each model, drug-binding pockets in the cavity forming part of the substrate permeation pathway were identified (see Table 2). The rigid docking procedures were performed using the Lamarckian genetic algorithm of Autodock 4.2 software. The optimized docking parameters were set as default values, except the number of runs which was 100. Torsionals in the residuals of binding pockets were not rotatable. A cluster analysis was performed on the docked results using an RMS tolerance of 2 Å. In each case the best docking result was considered as complex with the lowest binding energy. Hydrogen bindings between docked potent agents and related macromolecules were analyzed using ADT, the AutoDockTools program (ADT. Version 1.5.4) [95].

### 4.3. Similarity and Superimposition Study

The Tanimoto similarity coefficient (Tsc) for pairwise was computed as available on the ChemMine tools server (http://chemminetools.ucr.edu/ (accessed on 1 June 2021)) [30]. To obtain additional information concerning the shape of the investigated drug, the active conformations were chosen for superimposition. The atoms (except hydrogen atoms) common to these molecules were selected for the fitting procedure using SCIGRESS software, version 3.4.4, www.scigress.com. Their similarity was calculated as RMS fit. The RMS routine provided estimates of how closely molecules fit with each other. The lower the RMS value, the better the similarity [37].

## 5. Conclusions and Perspectives 

The mechanism of adult neurogenesis has not been fully understood and described so far. However, in light of current research, it has been found that it is initiated by an increase in the concentration of neurotransmitters in the brain. For this reason, it seems logical to suppose that drugs used in the pharmacotherapy of diseases with etiology resulting from a decreased level of neurotransmission with the participation of basic mediators (SER, NE, DA, GABA) in the nervous system may potentially act in the neurogenic mechanism. Undoubtedly, depression is one of these diseases, and PRX, as the most widely used antidepressant, meets the requirements of the research assumption. It should be emphasized that PRX belongs to the group of the most studied molecules. For this reason, we have a lot of well-documented data that can be used in the context of analyzing new mechanisms of action.

The study presented in this publication predominantly was meant to answer the question about the possibility of PRX acting in the neurogenic mechanism. Data from molecular studies indicate a potential neurogenic effect of PRX, which may be mainly due to the strong inhibition of hSERT. In order to identify the causes of different values of the inhibition parameters (pK_i_ and pIC_50_), it was reasonable to compare the structural similarity of the analyzed structures of the analyzed objects. First, the structural similarity of four basic neurotransmitters: SER, NE, DA and GABA and the corresponding targets, i.e., MAT and GAT1 transporters were analyzed, using the results of research based on molecular modeling techniques.

Using various techniques such as the Tanimoto test and overlap tests, it was shown that some structural similarities were found in the tested carriers and their transporters. Nevertheless, the analysis of structural similarities calculated as RMS determined by the superposition of the structures of two proteins considering the alpha carbon atoms of the respective amino acids seems to be crucial for understanding the interactions of various compounds with the analyzed targets. These results, in contrast to the data routinely presented in the literature, based on the amino acid sequence identification technique, highlight the structural differences of the analyzed transporters. These differences are of particular importance within the ligand binding site. The information obtained from this study indicates significant differences between MAT and GAT1, which may explain the low GAT1-3 and BGT1 inhibition values (potency and affinity, respectively) by PRX.

An additional, but very important, prospective achievement of the research carried out is the confirmation of some activity of PRX towards hGAT. This enables the development of research based on the modification of the PRX structure in order to increase the affinity for particular types of hGAT.

## Figures and Tables

**Table 1:** The summary of hSERT, hDAT, hNET, hGAT1 and PRX, neurotransmitters docking experiment results.

Complex		E_B_	pK_i_	Amino Acid Residues	H_B_		Angle	L_HB_	E_HB_
Protein	Ligand	kcal/mol			Donor	Acc	θ	Å	kcal/mol
hSERT	PRX	−13.92	10.20	ASP98	%NH2+	# COO	139.95	2.76	−1.68
	SER	−9.7	7.11	ALA96	%NH1+	# CONH	129.52	1.91	−0.27
				ASP98	%NH2+	# COO	132.83	1.78	−0.15
				SER336	%NH3+	# OH	164.35	1.86	−2.54
				SER438	%IndOH	# CONH	138.77	1.92	−0.27
	R−NE	−5.45	3.99	ASP98	%OH	# COO	134.19	1.81	−0.12
				PHE335	%NH3+	# CONH	174.95	2.09	−3.46
				GLU493	%m-Ph-OH	# COO	134.94	1.85	−0.05
	S−NE	−5.31	3.89	TYR95	# Ph-OH	%m-PH-OH	156.36	1.77	−5.21
				ASP98	%NH3+	# COO	147.64	1.81	−1.97
				ASP98	%OH	# COO	141.19	1.64	−6.26
	DA	−5.45	3.99	PHE335	%NH3+	# CONH	159.93	2.11	−0.87
				GLU493	%p-Ph-OH	# COO	154.89	1.96	−2.34
				GLU493	%m-PH-OH	# COO	155.26	2.01	−5.80
	GABA	−6.77	3.86	LYS490	# NH1+	%COO1	150.89	1.68	−0.62
				LYS490	# NH2+	%COO2	126.72	2.24	−0.05
hDAT/cocaine	PRX	−8.35	6.12	ASP476	%NH2+	# COO	144.54	2.64	−0.02
	SER	−7.77	5.70	ASP79	%NH1+	# COO	142.74	1.99	−0.12
				ASN157	%IndOH	# CONH2	120.70	2.17	−0.23
				SER422	%NH2+	# CONH	135.29	2.03	−2.57
	R−NE	−6.01	4.40	PHE76	%OH	# CONH	154.98	2.12	−1.02
				ALA77	%NH3+	# CONH	139.52	1.85	−1.81
				ASP79	%NH3+	# COO	149.09	1.81	−0.38
				ASP79	%m-PH-OH	# COO	145.33	2.15	−1.00
	S−NE	−6.01	4.41	ASP79	%p-PH-OH	# COO	168.08	1.89	−3.85
				ASP79	%m-PH-OH	# COO	153.47	1.73	−1.02
				ASP476	%OH	# COO	122.98	1.95	−0.26
				ASP476	%NH3+	# COO	127.31	2.12	−0.01
				ASP476	%NH3+	# COO	138.16	1.78	−0.12
	DA	−7.22	5.29	ASP79	%NH1	# COO	131.46	2.12	−2.93
				SER149	%p-Ph-OH	# CONH	158.76	2.19	−3.40
				TYR156	%NH2	# Ph-OH	159.81	2.12	0.032
	GABA	−4.62	3.38	ASP79	%NH1+	# COO	144.11	1.74	−1.10
hDAT/clomipramine	PRX	−8.02	5.88	ASP476	%NH2+	# COO	155.89	2.75	−0.04
	SER	−7.05	5.17	ASP476	%NH1+	# COO	124.04	2.19	−0.08
				ASP476	%NH2+	# COO	141.21	1.87	−0.51
				ASP476	%IndOH	# CONH	165.74	1.83	−4.42
	R−NE	−6.04	4.43	ASP385	%p-Ph-OH	# COO	159.51	1.77	−0.46
				ASP385	%m-PH-OH	# COO	171.74	1.96	−2.65
				ASP476	%OH	# COO	156.16	1.96	−1.07
	S−NE	−6.07	4.45	ASP385	%p-Ph-OH	# COO	160.65	1.83	−1.50
				ASP385	%m-PH-OH	# COO	158.36	2.11	−1.50
				ASP476	%OH	# COO	168.50	1.84	−1.29
	DA	−6.72	4.92	ASP385	%p-Ph-OH	# COO	156.93	1.87	−1.38
				ASP385	%m-PH-OH	# COO	162.14	2.00	−4.85
				ASP476	%NH3+	# COO	127.69	1.91	−0.10
	GABA	−4.34	3.18	ASN93	%NH1+	# CONH2	126.49	1.99	−0.85
				SER309	%NH2+	# OH	137.67	2.15	−2.84
hNET	PRX	−10.38	7.61	ALA73	%NH1+	# CONH	125.52	2.73	−1.06
				ASP75	%NH2+	# COO	162.17	2.74	−0.12
	SER	−5.91	4.33	PHE72	%NH1+	# CONH	152.37	2.18	−4.06
				ASP75	%NH2+	# COO	142.29	2.02	−0.04
				SER419	%NH3+	# OH	135.90	1.96	−2.85
				SER420	%IndOH	# OH	168.89	2.20	−4.18
	R−NE	−5.62	4.12	ASP75	%p-Ph-OH	# COO	170.95	1.72	−3.11
				ASP75	%m-PH-OH	# COO	153.61	1.74	−1.64
				ASP473	%OH	# COO	143.25	2.01	−0.01
	S−NE	−7.75	5.59	PHE72	%NH1+	# CONH	121.16	2.06	−0.56
				ASP75	%NH2+	# COO	153.53	2.19	−0.53
				SER318	%OH	# CONH	135.06	1.94	−1.34
				LEU319	%m-PH-OH	# CONH	131.15	2.14	−0.61
	DA	−7.25	5.31	ASP75	%NH1+	# COO	121.36	1.99	−0.13
				ALA145	%p-Ph-OH	# CONH	134.7	2.11	−0.93
				TYR152	%NH2+	# Ph-OH	169.39	2.18	−3.64
				SER419	%NH3+	# OH	139.35	2.25	−2.08
	GABA	−4.51	3.31	ASP75	%NH1+	# COO	151.09	1.81	−1.60
hGAT1	PRX	−5.93	4.35	ASP451	%NH2+	# CONH	151.56	2.91	−3.70
				ASP451	%NH2+	# COO	147.39	2.93	−3.13
	SER	−4.86	3.56	TYR139	%NH1	# Ph-OH	172.42	1.99	−5.78
				ASP451	%NH2	# COO	143.11	1.86	−2.83
				ASP451	%NH3	# CONH	122.09	1.87	0.023
				SER456	%m-PH-OH	# CONH	139.31	2.06	−2.94
				MET458	# CONH	%m-PH-OH	150.79	1.90	−4.45
				SER459	# CONH	%m-PH-OH	164.48	2.08	−5.04
	R−NE	−5.28	3.87	TYR139	%NH1	# Ph-OH	130.79	2.21	−1.60
				ASP451	%NH2	# CONH	148.66	1.85	−1.96
				MET458	# CONH	%p-PH-OH	163.77	1.99	−5.67
				SER459	# CONH	%p-PH-OH	151.54	1.93	−4.51
				LEU460	# CONH	%m-PH-OH	151.03	2.24	−2.73
	S−NE	−5.28	3.86	ASP451	%NH1	# COO	135.12	1.76	−0.47
				ASP451	%OH	# CONH	171.27	1.65	−2.93
				SER456	%m-PH-OH	# CONH	152.49	2.06	−3.09
				MET458	# CONH	%m-PH-OH	153.65	1.78	−4.80
				SER459	# CONH	%m-PH-OH	162.42	2.005	−5.5
	DA	−4.85	3.55	ASP451	%NH1	# COO	123.39	1.941	−0.503
				ASP451	%NH2	# CONH	127.83	2.017	−1.36
				SER456	%m-PH-OH	# CONH	172.05	1.937	−4.819
				MET458	# CONH	%m-PH-OH	151.59	2.026	−4.052
				SER459	# CONH	%m-PH-OH	162.66	2.124	−4.493
				LEU460	# CONH	%p-PH-OH	155.46	1.797	−5.329
	GABA	−6.24	4.49	ASP451	%NH1+	# COO	142.52	2.503	−0.026
				ASP451	%NH2+	# CONH	156.84	2.451	−3.32

H_B_—hydrogen bond, Acc—hydrogen bond acceptor, Hydrogen bond components: from the ABX % and from the protein #, E_B_—complex energy binding, θ—hydrogen bond angle, L_HB_—hydrogen bond length, E_HB_—hydrogen bond energy, pKi—calculated binding affinity.

**Table 2:** The composition of the binding pocket of the analyzed channel models: hSERT, hDAT, hNET, hGAT1.

Protein	Residues
hSERT	Tyr95, Ala96, Asp98, Gly100, Ala169, Ile172, Ala173, Tyr176, Phe335, Ser336, Gly338, Phe341, Val343, Ser438, Thr439, Ala441, Gly442, Val489, Lys490, Glu493, Glu494, Thr497, Gly498, Pro499, Leu502 [6]
hNET	Ala145, Tyr151, Ile315, Phe316, Ser420, Ala426 [44].
hDAT	Ala81, Tyr88, Asp385, Asp385, Asp476, Ala480, Phe472, Thr473, Asp476, His477, Ala480, Gly481, Thr482, Leu485 [6]
hGAT1	Tyr60, Ala61, Gly63, Gly65, Leu136, Tyr140, Phe294, Ser295, Tyr296, Gly297, Leu300, Thr400 [45]

**Table 3:** Inhibitory activity of PRX at hGAT1-3 and hBGT1 stably expressed in CHO cells using the [^3^H]GABA uptake assay.

IC_50_ (pIC_50_ ± S.E.M.) (µM)				
	hGAT1	hGAT2	hGAT3	hBGT1
PRX	256.1 (3.6 ± 0.03)	122.4 (3.9 ± 0.08)	110.6 (4.0 ± 0.04)	85.6 (4.1 ± 0.05)

## Data Availability

Not applicable.

## Abbreviations

θ	hydrogen bond angle
#	hydrogen bond components: from the protein
%	hydrogen bond components: from the ligand
acc	hydrogen bond acceptor
ADT	AutoDockTools
CHO	Chinese hamster ovary
CNS	central nervous system
CSF	cerebrospinal fluid
DA	dopamine
DAT	proteins of dopamine transporter
E_B_	complex energy binding
E_HB_	hydrogen bond energy
GABA	γ-aminobutyric acid
GAT1	GABA transporter isoform 1
GAT2	GABA transporter isoform 2
GAT3	GABA transporter isoform 3
GDA	generalized anxiety disorder
hSERT	human proteins of serotonin transporter
hDAT	human proteins of dopamine transporter
hNET	human proteins of norepinephrine transporter
hGAT1	human GABA transporter isoform 1
hGAT2	human GABA transporter isoform 2
hGAT3	human GABA transporter isoform 3
hBGT1	human betaine/GABA transporter
L_HB_	hydrogen bond length
MAT	monoamine transporters
MDD	major depressive disorder
NE	norepinephrine
NET	proteins of norepinephrine transporter
NPC	neural progenitor cells
NSC	neural stem cells
OCD	obsessive-compulsive disorder
PD	panic disorder
PDB ID	Protein Data Bank Identifier
pIC_50xxx_	inhibitory potency of xxx transporters
pK_ixxx_	binding affinity of xxx transporters
PMDD	premenstrual dysphoric disorder
PRX	paroxetine
PTSD	post traumatic stress disorder
R/S-NE	R/S enantiomers norepinephrine
RMS	root mean square
SAD	social anxiety disorder
SER	serotonin
SERT	proteins of serotonin transporter
SNRI	serotonin norepinephrine reuptake inhibitors
SSRI	selective serotonin reuptake inhibitors
TCA	tricyclic antidepressants
Tsc	Tanimoto similarity coefficient
TM	transmembrane segments
TMH	transmembrane segments helix
